# Numerical and Experimental Investigation of Novel Blended Bifurcated Stent Grafts with Taper to Improve Hemodynamic Performance

**DOI:** 10.1155/2018/8054850

**Published:** 2018-09-09

**Authors:** Ming Liu, Zhenze Wang, Anqiang Sun, Xiaoyan Deng

**Affiliations:** ^1^Key Laboratory for Biomechanics and Mechanobiology of Ministry of Education, School of Biological Science and Medical Engineering, Beihang University, Beijing 100083, China; ^2^National Research Center for Rehabilitation Technical Aids, Beijing Key Laboratory of Rehabilitation Technical Aids for Old-Age Disability, Key Laboratory of Rehabilitation Technical Aids Technology and System of the Ministry of Civil Affairs, No. 1 Ronghuazhong Road, Beijing BDA, Beijing 100176, China; ^3^Beijing Advanced Innovation Centre for Biomedical Engineering, Beihang University, Beijing 100083, China

## Abstract

The typical helical flow within the human arterial system is widely used when designing cardiovascular devices, as this helical flow can be generated using the “crossed limbs” strategy of the bifurcated stent graft (BSG) and enhanced by the tapered structure of arteries. Here, we propose the use of a deflected blended bifurcated stent graft (BBSG) with various tapers, using conventional blended BSGs with the same degree of taper as a comparison. Hemodynamic performances, including helical strength and wall shear stress- (WSS-) based indicators, were assessed. Displacement forces that may induce stent-graft migration were assessed using numerical simulations and in vitro experiments. The results showed that as the taper increased, the displacement force, helicity strength, and time-averaged wall shear stress (TAWSS) within the iliac grafts increased, whereas the oscillating shear index (OSI) and relative residence time (RRT) gradually decreased for both types of BBSGs. With identical tapers, deflected BBSGs, compared to conventional BBSGs, exhibited a wider helical structure and lower RRT on the iliac graft and lower displacement force; however, there were no differences in hemodynamic indicators. In summary, the presence of tapering facilitated helical flow and produced better hemodynamic performance but posed a higher risk of graft migration. Conventional and deflected BBSGs with taper might be the two optimal configurations for endovascular aneurysm repair, given the helical flow. The deflected BBSG provides a better configuration, compared to the conventional BBSG, when considering the reduction of migration risk.

## 1. Introduction

Bifurcated stent grafts (BSG) have been widely used in endovascular aneurysm repair (EVAR) for treating patients with abdominal aortic aneurysms (AAAs) [1]. During the EVAR procedure, BSG deployment becomes even more complicated and time-consuming if patients have abnormal AAA features; for example, the aneurysm neck is unfavorable, or the common iliac arteries are highly splayed [2]. To resolve these problems, the deployment technique of intentionally crossing the limbs of the BSG is regularly used [2–4]. With this deployment strategy, the cannulation time and postoperative complication rate in the face of atypical anatomy could be reduced significantly [2, 4, 5].

The typical helical flow observed within the human arterial system has significant physiological effects. The helical flow protects the arterial wall against atherosclerosis and thrombosis formation by affecting the transport of atherogenic lipids [6, 7] and reducing platelet adhesion on the arterial walls [8]. Because of these hemodynamic benefits of helical flow, it has been applied by researchers to design vascular devices to avoid intimal hyperplasia and thrombus formation, which are often caused by an unfavorable hemodynamic environment [9]. In clinical applications, the “crossed limbs” strategy might generate helical flows within the limb of crossed stent grafts [3, 5], which is believed to be advantageous in AAA treatment. One significant feature of the arterial system that needs to be addressed is its taper, or the decreasing diameter of an artery as it becomes an arteriole. Our previous studies revealed that the aortic taper was capable of stabilizing blood flow and maintaining helical flow strength, which evidently reduced the accumulation of low-density lipoproteins on the aortic luminal surface [7]. Accordingly, the taper is of great significance for designing vascular devices.

Optimal design of the stent graft is important for improving its hemodynamic performance. For instance, the blended bifurcated stent graft (BBSG) was proposed by Morris et al.; this graft eliminates the sudden decrease in cross-sectional area at the bifurcation [10]. This type of BBSG is believed to improve hemodynamic performance and provide better long-term outcomes. In this study, the deflected BBSG was proposed based on the specific configuration, including the “crossed limbs” strategy and the BBSG, proposed by Morris et al. [5, 10]. Furthermore, the taper configuration was added to both the conventional and deflected types of BBSGs to obtain the desired high helical flow. In order to assess these new conceptual designs, we analyzed their hemodynamic performance by analyzing numerical simulations. To analyze the displacement force that may induce stent-graft migration, numerical simulation and in vitro experiments were performed to evaluate the migration risk.

## 2. Methods and Materials

### 2.1. Geometry

**Figure 1:**
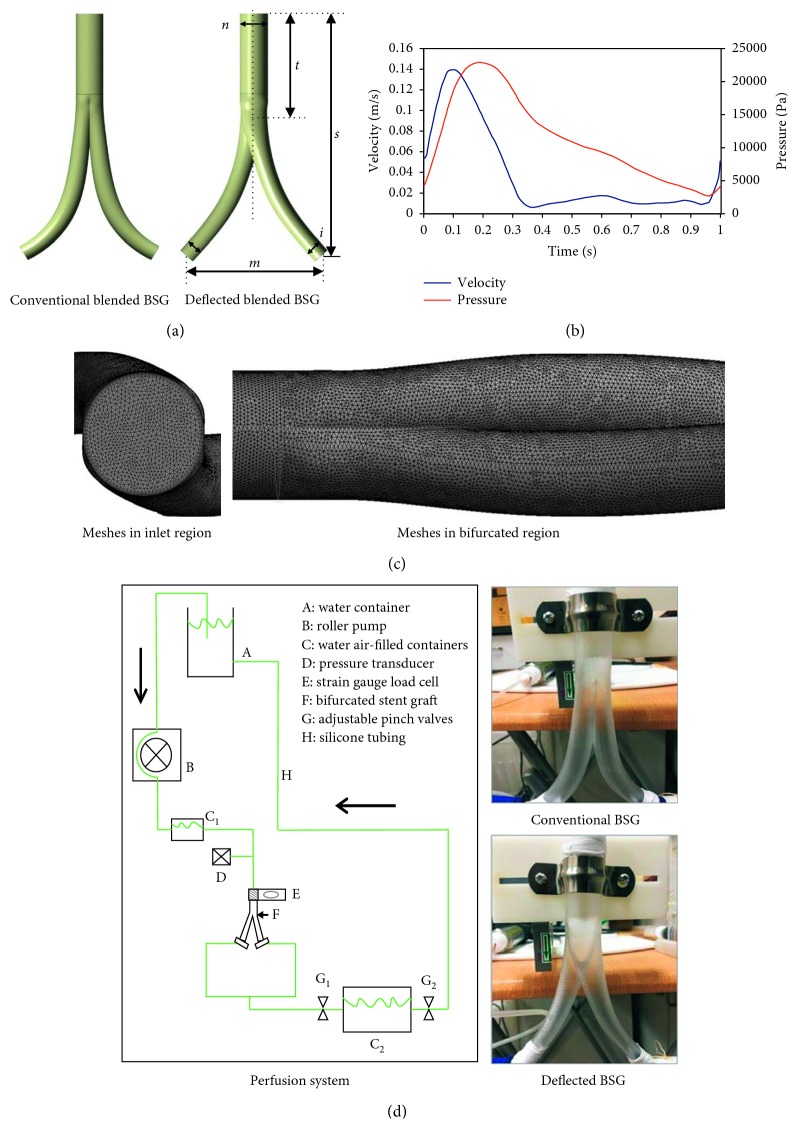
(a) Geometrical models of the ideal conventional and deflected types of blended bifurcated stent grafts (BBSGs) with taper to different degrees. (b) Imposed inlet velocity and outlet pressure waveforms. (c) Mesh presentation in the inlet and bifurcated regions. (d) Schematic presentation of the perfusion system and two types of BBSGs made of semitransparent photosensitive resin created with laser rapid prototyping technology.

As depicted in Figure 1(a), referring to the BBSG parameters described in the previous literature [11, 12], two series of geometrical models with taper features, in terms of conventional and deflected BBSGs, were generated using SolidWorks (SolidWorks Corp, Concord, MA, USA). The conventional BBSG was designed based on the common BSG with blended features in the bifurcated region, which eliminates the sudden decrease in cross-sectional area at the bifurcation, as proposed by Morris et al. [10]. The deflected BBSG was designed based on the “crossed limbs” deployment strategy. When using the “crossed limbs” strategy in EVAR, the iliac grafts usually get crossed, which generates the helical flow [2, 5]. The differences between the conventional and novel designed deflected BBSGs are that the division surface of the bilateral iliac grafts for conventional BBSG is located in the symmetry plane of the iliac grafts, whereas the division surface of the bilateral iliac grafts for deflected BBSG is located in the base plane of the iliac grafts. The trunk body of all BBSG configurations was kept identical. The overall length (*s*) was 154.64 mm in the axis direction, and the trunk BSG (*t*) was 71 mm. The side length (*m*) was 82 mm. The trunk of the BBSG was 17 mm in diameter (*n*). Regarding the taper feature, it was set as the internal diameter, and it decreased progressively from the bifurcation region (*n*) to the outlet (*i*). For conventional and deflected types of BBSG, three grafts with differing tapers were constructed. Therefore, six models were constructed in total; among these models, conventional BBSGs were denoted as (a1) tapered 17–10 mm BBSG, (a2) tapered 17–9 mm BBSG, and (a3) tapered 17–8 mm BBSG, whereas deflected BBSGs were denoted as (b1) tapered 17–10 mm BBSG, (b2) tapered 17–9 mm BBSG, and (b3) tapered 17–8 mm BBSG. For instance, the tapered 17–10 mm graft had a bifurcation region diameter of 17 mm and an outlet diameter of 10 mm.

### 2.2. Governing Equations

Numerical simulations were created on the basis of the three-dimensional (3D), incompressible Navier–Stokes in Equation (1) and continuity equations in Equation (2) as expressed below:

(1) $$\begin{array}{c} \rho \left(\frac{\partial \vec{\nu }}{\partial t}+\left(\vec{\nu }\cdot \nabla \right)\vec{\nu }\right)=-\nabla p+\nabla \tau , \end{array}$$

(2) $$\begin{array}{c} \nabla \cdot \vec{\nu }=0, \end{array}$$

where $\vec{\nu }$ denotes the fluid velocity vector and *p* refers to the pressure. *ρ* denotes the blood density (*ρ* = 1050 kg/m^3^), and *τ* denotes the stress tensor:

(3) $$\begin{array}{c} \tau =2\eta \left(\dot{\gamma }\right)D, \end{array}$$

where **D** denotes the respective deformation tensor, $\dot{\gamma }$ refers to the shear rate, and *η* indicates the blood viscosity as a function of the shear rate. **D**(**u**)=(1/2)(∇**u**+∇**u**^*T*^) represents the strain rate tensor, and $\dot{\gamma }=\sqrt{2D:D}$ is the strain rate tensor modulus.

The non-Newtonian characteristic of the blood flow was factored in with the Carreau model expressed as follows [13]:

(4) $$\begin{array}{c} \eta \left(\dot{\gamma }\right)=\eta_{\infty }+\left(\eta_{0}-\eta_{\infty }\right){\left[1+{\left(\lambda \dot{\gamma }\right)}^{2}\right]}^{\left(\left(n-1\right)/2\right)}, \end{array}$$

where *η*_0_ denotes the zero shear rate viscosity, *η*_*∞*_ denotes the infinite shear rate viscosity, and *λ* refers to the relaxation time constant. The Carreau model was well matched with the experimental data [14], as found, where *η*_*∞*_ = 3.45×10^−3^ Pa·s, *η*_0_ = 5.6×10^−2^ Pa·s, *n* = 0.3568, and *λ* = 3.313 s.

### 2.3. Boundary Conditions and Meshing

Steady flow was first simulated; subsequently, the solution of steady simulation was employed as the initial condition to further simulate the pulsatile flow. An average velocity of 0.044 m/s was extracted from the velocity waveform in Figure 1(b), and a constant of 13300 Pa was adopted at the inlet and outlets to simulate steady flow. The inlet of the BBSG corresponded to the suprarenal aorta entrance, and the outlets corresponded to the iliac arteries outflow tracts. The Reynolds number used for the steady condition was 204, and the convergence threshold value was 1 × 10^−5^. The waveform of time-dependent flat flow velocity presented in Figure 1(b) was set at the inlet, and the waveform of time-dependent pressure boundary was assigned at the outlets to simulate pulsatile flow [15]. The BBSG wall was regarded as rigid and nonslippery [16].

Using ANSYS (ANSYS Inc., Canonsburg, PA), tetrahedral volume meshes were generated for these models. To ensure that our results were mesh independent, three additional mesh sizes were generated and employed to perform the calculations. When the averaged difference in the velocity and pressure of the left outlet between two successive simulations was less than 1% under steady flow (Table 1), mesh independence was considered to be achieved. The meshes that met the standard were directly used to perform the pulsatile simulations. The final volume of the meshes was nearly 1.7 million cells for each model, as shown in Figure 1(b). Details of the mesh-independent study are provided in Table 1, which shows the velocity and pressure differences between consecutive meshes.

### 2.4. Numerical Scheme

By employing ANSYS Fluent, the numerical simulations were performed by adopting 200 steps per cycle, with step times of 0.005 s [11]. A pressure-based solver was employed with a second-order upwind scheme to spatially discretize the momentum. The residual values for continuity and velocity were set as 1.0 × 10^−5^ [17]. In total, five periods were computed to ensure that the solutions were periodic. The solutions of the final period were extracted and analyzed using the MATLAB and Tecplot software.

### 2.5. Quantities of Interest

To better understand the diagnostic and therapeutic aspects of blood flow for treating arterial diseases, numerical simulation studies on hemodynamic effect of blood flow have been widely performed [18, 19]. By simplifying the blood characteristics, several WSS-based descriptors including WSS, time-averaged wall shear stress (TAWSS), oscillating shear index (OSI), and the relative residence time (RRT) could be calculated from the solutions of the simulations. These WSS-based descriptors might represent different hemodynamic disturbances and would present different impacts on endothelial cell homeostasis and platelet activation/aggregation. Therefore, they could potentially be used to predict the representative regions that have a high occurrence risk of stenosis and aneurysm in arteries such as the coronary artery, renal artery, or aorta or occlusion of bypass graft [9, 20].

The helicity can be employed to measure the alignment or misalignment of the local velocity and vorticity vectors, and the rotating direction of the helical structures was indicated by the sign of helicity [21]. The helicity density *H*_d_ was defined using the following equation[19, 22]:

(5) $$\begin{array}{c} H_{\text{d}}=\vec{\nu }\cdot \left(\nabla \times \vec{\nu }\right)=\vec{\nu }\cdot \omega , \end{array}$$

By adopting the TAWSS, the WSS on the BSG during the whole cardiac cycle was assessed:

(6) $$\begin{array}{c} \mathrm{TAWSS=}\frac{1}{T}\int_{0}^{T}\left|\mathrm{WSS}\left(s,t\right)\right|\;dt, \end{array}$$

where *T* denotes the time and *s* refers to the position on the stent-graft wall.

The OSI was employed to uncover the directional variation of the WSS vector in a pulsatile cycle; a higher value occurred particularly in regions characterized by disturbed flow. This was expressed as follows [23]:

(7) $$\begin{array}{c} \text{OSI}=\frac{1}{2}\left[1-\left(\frac{\left|\int_{0}^{T}\mathrm{WSS}\left(s,t\right)\;dt\right|}{\int_{0}^{T}\left|\mathrm{WSS}\left(s,t\right)\right|dt}\right)\right]. \end{array}$$

RRT is employed for assessing the resident time of blood flow [24] and defined as

(8) $$\begin{array}{c} \text{RRT}=\frac{1}{\left(1-2\times \mathrm{OSI}\right)\times \mathrm{TAWSS}}. \end{array}$$

## 3. Results

### 3.1. Flow Pattern

**Figure 2:**
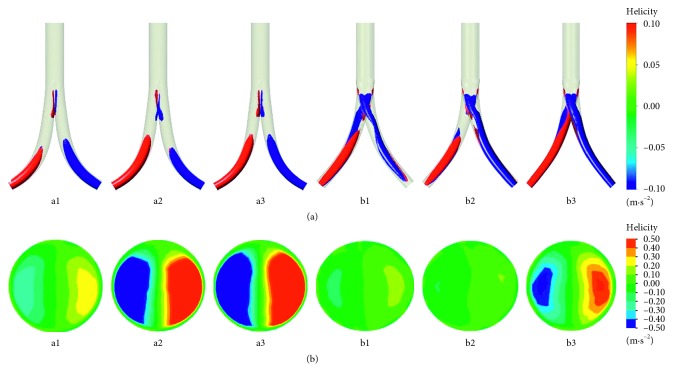
(a) Helicity isosurfaces of −0.5/0.5 ms^−2^ under steady-state simulation. (b) Surface contours of helicity at the left iliac graft outlet steady-state simulation. The right-handed helical structure with positive value was colored red, while the left-handed helical structure with negative value was colored blue.

The helicity isosurfaces of −0.5/0.5 m·s^−2^ are presented in Figure 2. As shown in Figure 2, two helical flow structures widely exist along the iliac grafts from the bifurcation region. When the conventional BBSG was observed, the helical flow structures were initially generated form the middle portion of the iliac graft and developed until the outlets. In comparison, the helical flow structures in the deflected BBSG were initially generated from the bifurcated region and developed along the iliac graft until the outlets. The helical flow structures within the deflected BBSG were much more obvious than that in the conventional BBSG. As depicted in Figure 2, double-helical flows can be observed at the left iliac graft outlet. As the taper increases, these helical flows become even more apparent. The absolute helicity at the iliac outlet gradually increased from 0.11 to 0.51 m·s^−2^, until it finally reached 0.63 m·s^−2^ as the taper for the conventional BBSG increases. The absolute helicity at the left iliac outlet gradually increased from 0.05, to 0.04, to 0.21 m·s^−2^ for the deflected BBSG with the increasing taper.

**Figure 3:**
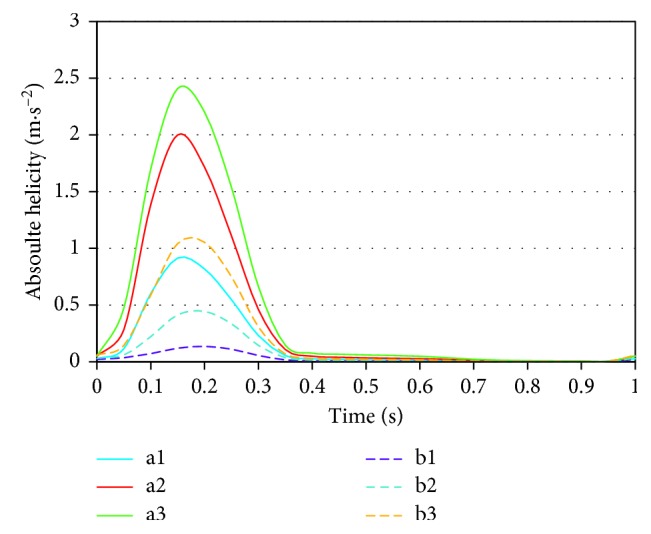
Absolute helicity of the left outlet in the pulse cycle during the pulsatile calculations.

The varying trends of absolute area-averaged helicity for the left outlet during the pulsatile calculations are plotted and compared in Figure 3. The absolute helicity in systole evidently surmounts the rest of the cycle. In diastole, helicity remains basically the same and approaches zero. As the taper increased, the peak absolute area-averaged helicity progressively increased from 0.8 m·s^−2^ (a1) to 2.2 m·s^−2^ (a3) for the conventional BBSG. Yet the peak absolute area-averaged helicity increases from 0.14 m·s^−2^ (b1) to 1.05 m·s^−2^ (b3) for the deflected BBSG as the taper increases.

### 3.2. Hemodynamic Indicators

**Figure 4:**
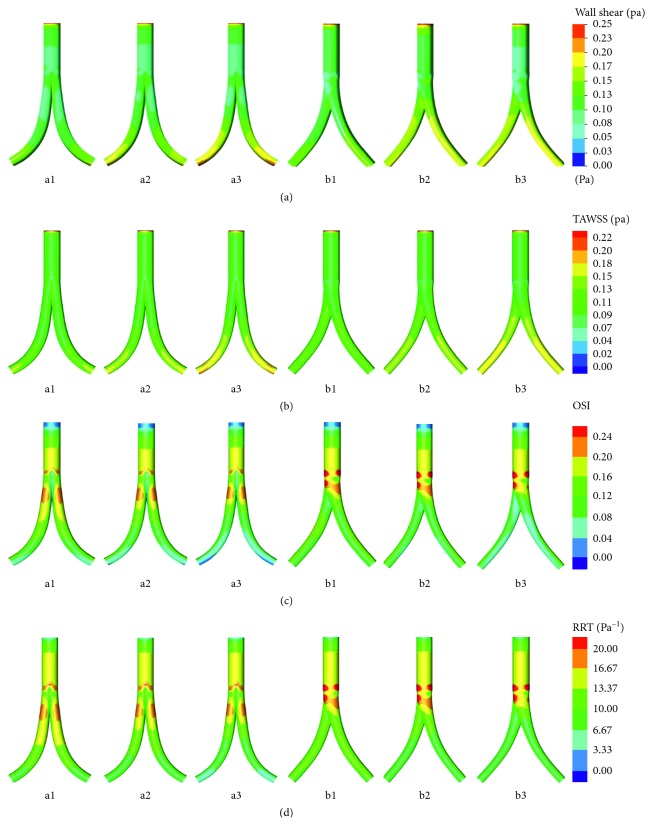
Distributions of hemodynamic indicators on the six models. (a) WSS contour; (b) TAWSS contour; (c) OSI contour; (d) RRT contour.

**Figure 5:**
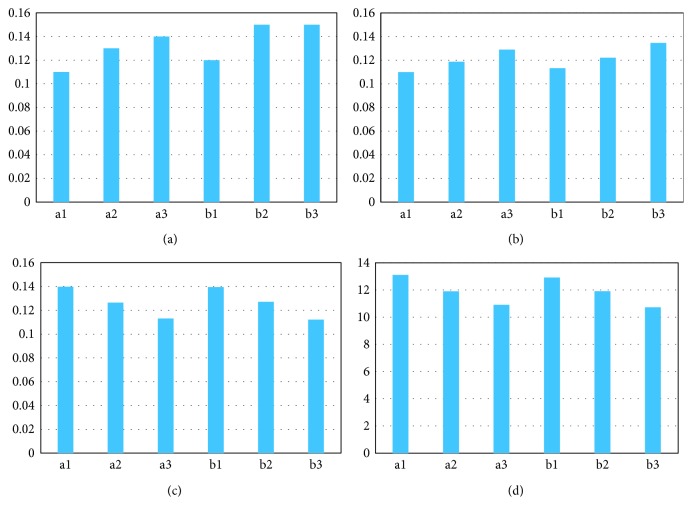
Area-weighted averages of hemodynamic indices in the iliac grafts of the six models. (a) WSS (Pa); (b) TAWSS (Pa); (c) OSI; (d) RRT (Pa^−1^).

As observed in Figure 4, the comparatively high WSS areas were observed in iliac grafts, especially in the distal parts of the iliac grafts. The comparatively high WSS areas become progressively apparent as the taper increased. The area-averaged weighted WSS distribution of the iliac grafts was compared using a histogram (Figure 5). As the taper increased, the WSS gradually increased from 0.11 to 0.14 Pa for the conventional BBSG, and from 0.12 to 0.15 Pa, for the deflected BBSG.

As observed in Figure 4, the relatively high TAWSS areas could be observed in the distal parts of the iliac grafts. As the taper increased, the relatively high TAWSS areas become even more obvious. The area-averaged weighted TAWSS distribution of the iliac grafts was compared using a histogram (Figure 5). As the taper increased, the TAWSS in the iliac grafts increased from 0.11 to 0.13 Pa for the conventional BBSG and increased from 0.11 to 0.13 Pa for the deflected BBSG. TAWSS on the iliac grafts showed no major differences between the conventional and deflected BBSG.

The scattered high OSI areas are observed in the bifurcation region, as presented in Figure 4. The low OSI area in the inner surface can be observed distributed on the distal parts of BBSG for the conventional BBSG, and the same phenomenon occurs in the deflected BBSG. Figure 5 depicts the OSI on the iliac grafts in the form of a histogram. As the taper increases, the area-averaged weighted OSI in the iliac grafts gradually decreases from 0.14 to 0.10 for the conventional BBSG and decreases from 0.14 to 0.11 for the deflected BBSG.

The scattered high RRT areas can be observed in the bifurcation region, as observed in Figure 4. The distal parts of BBSG have an apparent low RRT area for the conventional BSG, while the deflected BBSG also presents a low RRT area in the inner face of the distal parts. As shown in Figure 5, the area-averaged weighted RRT in the iliac grafts gradually decreases from 13.1 to 10.91 Pa^−1^ for the conventional BBSG with increasing taper, and from 12.92 to 10.72 Pa^−1^, for the deflected BBSG as with increasing taper.

### 3.3. Displacement Force

**Figure 6:**
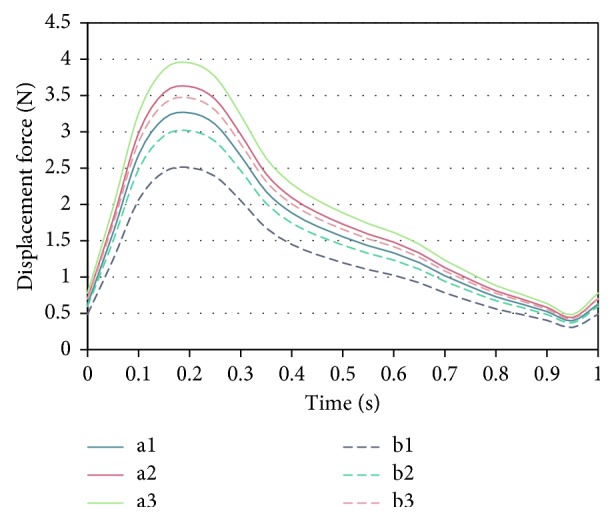
Variation trends of displacement force acting on the BBSG in the pulse cycle.

The displacement force likely to induce BSG migration was extracted and compared as variation trends. The displacement force peaked under the maximum pressure, as indicated in Figure 6. The displacement force progressively increased for both types of BBSGs as the degree of the taper increased. The peak displacement force for conventional BBSGs ranged from 3.25 N to 3.9 N and ranged from 2.5 N to 3.5 N for deflected BBSGs. The deflected BBSG clearly had a lower magnitude of displacement force in contrast to the conventional BBSG under the same taper. The peak displacement forces during the pulsatile cycle in both types of BBSG were extracted and compared. It was seen that the peak force in the deflected BBSG was significantly lower than that in the conventional BBSG (*p* < 0.05, *t*-test), showing that the deflected BBSG posed a lower risk of migration.

#### 3.3.1. In Vitro Experiment

To evaluate the migration risk of the newly designed BSG, we designed an experimental aortic perfusion system to measure the displacement force. As shown in Figure 1(d), the steady flow and aortic pressure within the BSG were provided by the perfusion system. A fluid mixture consisting of 33.3% by volume of glycerol in water was used to mimic blood. The mixed fluid used in the in vitro experiments has characteristics similar to those of blood, with a density of approximately 1050 kg/m^3^, and a dynamic viscosity of approximately 0.0035 Pa·s. A roller pump and silicon tubes were used to construct the fluid circuit. The fluid was perfused into the circuit at room temperature to resemble aortic perfusion. Peripheral resistance was achieved with a container and pinch valves. The pressure was regulated by adjusting the pinch valves and water level in the water/air-filled containers, which were located at the distal part of the circuit. The water/air-filled containers were placed after the roller pump to minimize the flow disturbances.

As depicted in Figure 1(d), all BBSG models used in the experiments were made of photosensitive resin created with laser rapid prototyping technology. The geometrical size of the cavity within the experimental models was exactly the same as that of the geometry model in the computational simulations. The wall thickness of the semitransparent model used in the in vitro experiment was 1 mm. These resin-transparent BBSG models were inserted into the circuit. The proximal part of the BBSG was anchored rigidly to the strain gauge load cells using connectors. The load cell was fixed strongly to ensure that it could sense the displacement changes of the BBSG. The load cell is one type of the weighing sensor that can be used to measure the force along the tension and compression directions. The rated load ranged from 0 to 10 N. The BBSG model was placed on the outside surface of the connectors to ensure that the load cell could fully receive the displacement force induced by the flow. The calibration was performed with weights. The proximal and distal parts of the BBSG were connected to the silicon tube with highly elastic soft rubber tubes to ensure that the measurements would not be influenced. For monitoring the perfusion in the iliac graft, the pressure transducer was deployed and inserted into the circuit. To record the displacement force values, the load cell was linked with a force monitor.

Measurements with both types of BBSG at various degrees of the taper were recorded at the perfusion pressures of 60, 80, and 100 mmHg. Before measurement, we conducted zero leveling and in situ calibration of perfusion pressure. When the flow remained stable, we started to perform the measurements every 20 seconds, after which the force value depicted was recorded 10 times in total. All measurements were performed under conditions of steady flow. The data of displacement force are presented in Table 2 in the format of mean ± SD (standard deviation)

As shown in Table 2, the displacement force gradually increases with the increasing pressure and the increasing degree of the taper. For the deflected BBSG, the displacement forces were smaller than those for the conventional BBSG under identical pressure. Compared with conventional BBSG, deflected BBSG showed a decrease in the displacement force between 0.07 N and 0.11 N in the cases of the three levels of perfusion pressure.

## 4. Discussion

Optimized design of a BSG is critical for improving its hemodynamic performance and preventing postoperative complications [10, 15, 25]. In this study, we proposed two series of conventional and deflected BBSG models with various degrees of the taper. The hemodynamic performance was assessed and compared by analyzing numerical simulations. The displacement force, which would influence the migration risk, was evaluated by conducting in vitro experiments.

Helicity density was used to qualify the helical flow within the BSG; the sign of helicity density represented the rotating direction of helical structures [19, 26]. As indicated by the simulation results, two dominant helical flows were observed in both the conventional and deflected BBSGs with higher tapers, and the helicity strength increased with an increase in the taper. Helical blood flows are believed to have physiological functions that protect the arteries by suppressing the accumulation of atherogenic low-density lipoproteins within the arterial wall [7], enhancing O_2_ supply to the artery [27], and reducing platelet and monocyte adhesions [8, 28]. Liu et al. investigated the hemodynamic performance within the helical graft by adding the taper feature [9]; their findings revealed that the taper feature enhanced the helical flow in the helical grafts, thus reducing the possibility of thrombus formation in the graft and improving the graft patency. The taper feature in our models had a similar effect. Therefore, the proposed blended grafts with taper involving conventional and deflected blended BSGs may decrease the possibility of thrombus formation within the stent grafts and prevent stent-graft failure in the long term. Especially, the helical structure within the deflected BBSG was continually maintained from the bifurcated region till the outlets, which is much wider than that seen in the conventional BBSG. Therefore, the deflected BBSG may provide a stronger preventive effect against the thrombosis formation within the iliac graft and reduce the occlusion risk of the stent graft.

The presence of the taper would increase both helicity and hemodynamic performance by elevating WSS and alleviating OSI and RRT in the iliac grafts for both types of BBSGs proposed in this work. It has been well established that low WSS and high OSI are of great significance for intimal hyperplasia, on the basis of their effects on the function of endothelial cells [29]. High OSI and RRT can result in thrombus formation in the way of stimulating platelet aggregation, enhancing the collision of activated platelets and increasing the residence time of procoagulant microparticles [30]. In this regard, the existence of the taper may decrease the risk of thrombus formation in the grafts. Our results indicated the taper would improve the hemodynamic in both conventional and deflected BBSGs. The BBSG showed a higher WSS and lower RRT on the iliac graft. Therefore, the deflected BBSG might provide a better hemodynamic performance than the conventional BBSG.

BSG migration remains a common complication after EVAR and could be impacted by various factors (e.g., the blood condition and configuration of BSG), as demonstrated by numerous researchers [11, 31]. When compared with the displacement force calculated from the simulation and the in vitro experiments, it was found that the variation trends of displacement force following the perfusion pressure consistently increased as the perfusion pressure increased. The changing trends of displacement force following the perfusion pressure were consistent with the results of the study by Li and Kleinstreuer [11]. Our results also showed that the displacement force would increase as the taper degree increased, when considering the taper feature. Roos et al. also investigated the effects of perfusion pressure and the taper feature on displacement force through in vitro experiments [32]. The displacement force waveforms were seen to have similar temporal behavior to those of the perfusion pressure waveform. They also found that the displacement force increased significantly for the tapered stent graft when compared with the nontapered stent graft. Our results obtained via computational simulations and in vitro experiments revealed that the displacement force would increase with the increase of the taper. Accordingly, the taper could also count as one factor that primarily affects the migration behavior of BBSGs as proposed in this paper. Our results also revealed that the displacement force was smaller in the deflected BBSG when compared with that in the conventional BBSG under identical taper conditions. In this regard, the migration risk assumed by the deflected BSG is smaller than that by the conventional BBSG. Thus, the deflected BBSG with taper might be the better optimized design for BBSG from the viewpoint of prohibiting the BSG migration.

In this study, idealized models with rigid walls were employed. As the hemodynamic effect was the main point, we intended to illustrate the characteristics of stent graft were not considered in our study. This could be one limitation of our study. These simplifications have been proposed as reasonable [9, 33] and provide clear comparisons, enabling a distinct conclusion; meanwhile, these simplified settings could be verified by conducting in vitro experiments. The biological response of platelets to the shear stress is not considered in the BBSG. The formation of thrombosis within the stent graft and the migration behavior of stent graft remain a gradual process. Therefore, a larger observational period is needed to detect the outcomes of endovascular aneurysm repair. Although potential risk of endograft migration and thrombosis can be evaluated using computational simulation and in vitro experiment, the conclusion is a preliminary conclusion and needs further study.

## 5. Conclusion

In summary, the present study revealed that novel BBSGs with taper, including conventional and deflected types, would generate helical flow and that the existence of the taper feature could enhance helical flow and hemodynamic performance. Therefore, the taper feature might serve as one optimal configuration for designing the BSG when considering the benefits of the helical flow mechanism. As the hemodynamic performance and migration risk must also be considered when optimizing the design of the BBSG, the risk of the deflected BBSG may be the better choice (when compared with the conventional BBSG) as it can provide better hemodynamic performance and poses a reduced risk of migration.

## Figures and Tables

**Table 1:** Mesh-independent study results.

Mesh cells	Mesh nodes	Outlet velocity difference (%)	Outlet pressure difference (%)
973,257	267,382		
1,242,534	332,187	0.05	0.05
1,457,826	397,248	0.03	0.04
1,723,820	462,861	0.01	0.01

**Table 2:** Displacement forces (N) acting on two types of blended bifurcated stent graft (BBSG) under various degrees of the taper.

	Conventional BBSG			Deflected BBSG		
Taper (mm)	17–10	17–9	17–8	17–10	17–9	17–8
60 (mmHg)	0.78 ± 0.01	0.82 ± 0.01	0.87 ± 0.01	0.71 ± 0.01	0.74 ± 0.01	0.78 ± 0.01
80 (mmHg)	0.93 ± 0.01	0.96 ± 0.01	1.01 ± 0.01	0.89 ± 0.01	0.92 ± 0.01	0.96 ± 0.01
100 (mmHg)	1.12 ± 0.03	1.17 ± 0.01	1.24 ± 0.01	1.03 ± 0.01	1.07 ± 0.01	1.13 ± 0.01

## Data Availability

The data used to support the findings of this study are included within the article.
